# An Unlikely Case of Blastomycosis in an Elderly Female: A Diagnostic Challenge in a Non-endemic Region

**DOI:** 10.7759/cureus.69276

**Published:** 2024-09-12

**Authors:** Natallia Machecha, Jaskomal Phagoora, Sonu Sahni, Luis F Meza

**Affiliations:** 1 Pulmonary, Critical Care, and Sleep Medicine, East Carolina University Brody School of Medicine, Greenville, USA; 2 Medicine, Touro College of Osteopathic Medicine, New York City, USA; 3 Pulmonary Medicine, Harlem Hospital Center, New York City, USA; 4 Research Medicine, New York Institute of Technology College of Osteopathic Medicine, New York City, USA; 5 Primary Care Medicine, Touro College of Osteopathic Medicine, New York City, USA

**Keywords:** chronic obstructive pulmonary disease (copd), pulmonary blastomycosis: adve, pulmonary infection, community-acquired pneumonia (cap), itraconazole treatment, systemic mycoses, clinical case report, fungal lung infection, non-endemic regions, blastomyces dermatitidis

## Abstract

*Blastomyces dermatitidis* is a fungus typically found in the soil of endemic regions such as the Midwest, concentrating in areas like Ohio, Mississippi, and the Great Lakes area. The systemic infection caused by inhaling *Blastomyces dermatitidis *is known as blastomycosis. The frequency of blastomycosis in non-endemic regions is increasing for a variety of speculated reasons, such as higher rates of immunosuppressed individuals and possible climate. Due to clinician unfamiliarity, misdiagnosis of blastomycosis is common, which potentiates worsening systemic infections. This study shows the clinical course of a patient with blastomycosis in a non-endemic region, highlighting the need for education for clinicians in non-endemic areas.

A 72-year-old female with a history of chronic obstructive pulmonary disease (COPD), coronary artery disease, a 47-year smoking history, and hypertension presented for outpatient management of COPD. CT three months prior to presentation showed nodular opacities in the lungs. A bronchoscopy was performed and revealed negative findings for malignancy or infection; the patient developed worsening symptoms leading to hospitalization. Subsequent testing revealed *Blastomyces dermatitidis*. She was promptly treated with a six to 12-month course of itraconazole with close follow-up. The study highlights the need not to rule out causes of infection based on location. Blastomycosis can resemble community-acquired pneumonia. Making the correct diagnosis is paramount, as delays can result in morbidity. Fungal cultures may be the gold standard, but due to the long culture time, there need to be other diagnostic tests like urine antigen testing. This study highlights the need to increase awareness of clinicians who experience blastomycosis patients in a non-endemic region.

## Introduction

*Blastomyces dermatitidis* is a fungus found in soil or decaying organic material. It is endemic in areas such as the Midwest, predominantly in Ohio, Mississippi, and the Great Lakes region [1]. Inhalation of the fungus is the predominant means of infection, which can lead to a systematic infection, blastomycosis [1]. The common presentation of this infection is pulmonary, resembling pneumonia of bacterial origin. Other systems that may be affected are the skin, central nervous system, bones, and genitourinary [1]. The severity of the disease varies based on the immune system of the individual, as immunocompromised individuals are at risk for disseminated infection [1]. Although *Blastomyces dermatitidis* is confined to endemic regions, reports of non-endemic blastomycosis are on the rise [2]. This condition may be misdiagnosed in non-endemic areas due to its non-specific symptoms, which may delay treatment and increase pathogenicity. This study highlights the clinical and diagnostic course of a patient with blastomycosis in a non-endemic region. Reporting such cases is paramount to educate clinicians in non-endemic areas and to ensure proper interventions for patients.

## Case presentation

A 72-year-old female with a past medical history of chronic obstructive pulmonary disease (COPD) GOLD category B on 2 L O_2_ at baseline, coronary artery disease, hypertension, and beta-lactam allergy presented to her outpatient pulmonology appointment to establish care and manage COPD. She recently moved from New York and endorsed a 47-year two-pack per day smoking history, but stated she quit 11 years ago. Imaging done three months prior demonstrated multiple nodular opacities in the right upper and right middle lobe as well as subcarinal adenopathy. *Mycobacterium avium *lung was suspected, so the patient was scheduled for an electromagnetic navigation bronchoscopy (ENB), bronchoalveolar lavage (BAL), and endobronchial ultrasound (EBUS) for further workup.

No features diagnostic of malignancy were observed in the fine-needle aspirate (FNA) or BAL. Moreover, no acid-fast or fungal organisms were identified. Lung parenchyma demonstrated reactive atypia and evidence of acute inflammation, and cultures obtained grew *Streptococcus viridans*. She completed a five-day course of levofloxacin and prednisone post-procedure.

In the days following the bronchoscopy, the patient developed a productive cough, weakness, and increased shortness of breath with intermittent fevers. Her symptoms progressively worsened, and 11 days after the bronchoscopy, the patient went to the ED for treatment. Initial workup demonstrated a leukocytosis of 21,200 cells with a neutrophilic predominance, procalcitonin elevates to 2.2 ng/mL, and scattered crackles through both left and right lung fields on physical examination. The infectious workup was negative for influenza A, B, and respiratory syncytial virus (RSV). CXR did not show focal consolidation, pneumothorax, or large pleural effusions. She was given a one-time azithromycin dose and started on a five-day moxifloxacin course for suspected underlying pneumonia.

**Figure 1:**
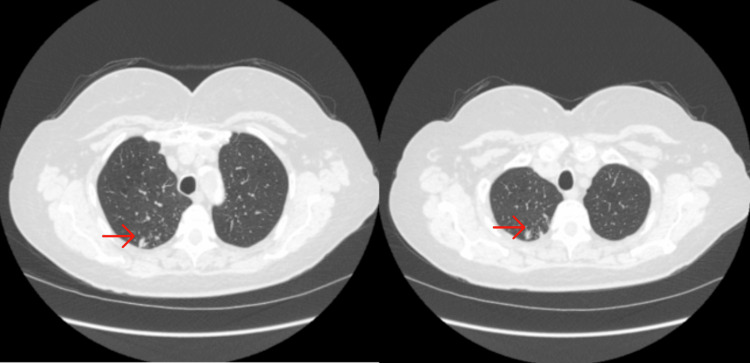
CT chest performed three months prior to the initial presentation to the Outpatient Department of East Carolina University Pulmonology Clinic demonstrating multiple RUL and RML nodular opacities. RUL: right upper lobe; RML: right middle lobe

**Figure 2:**
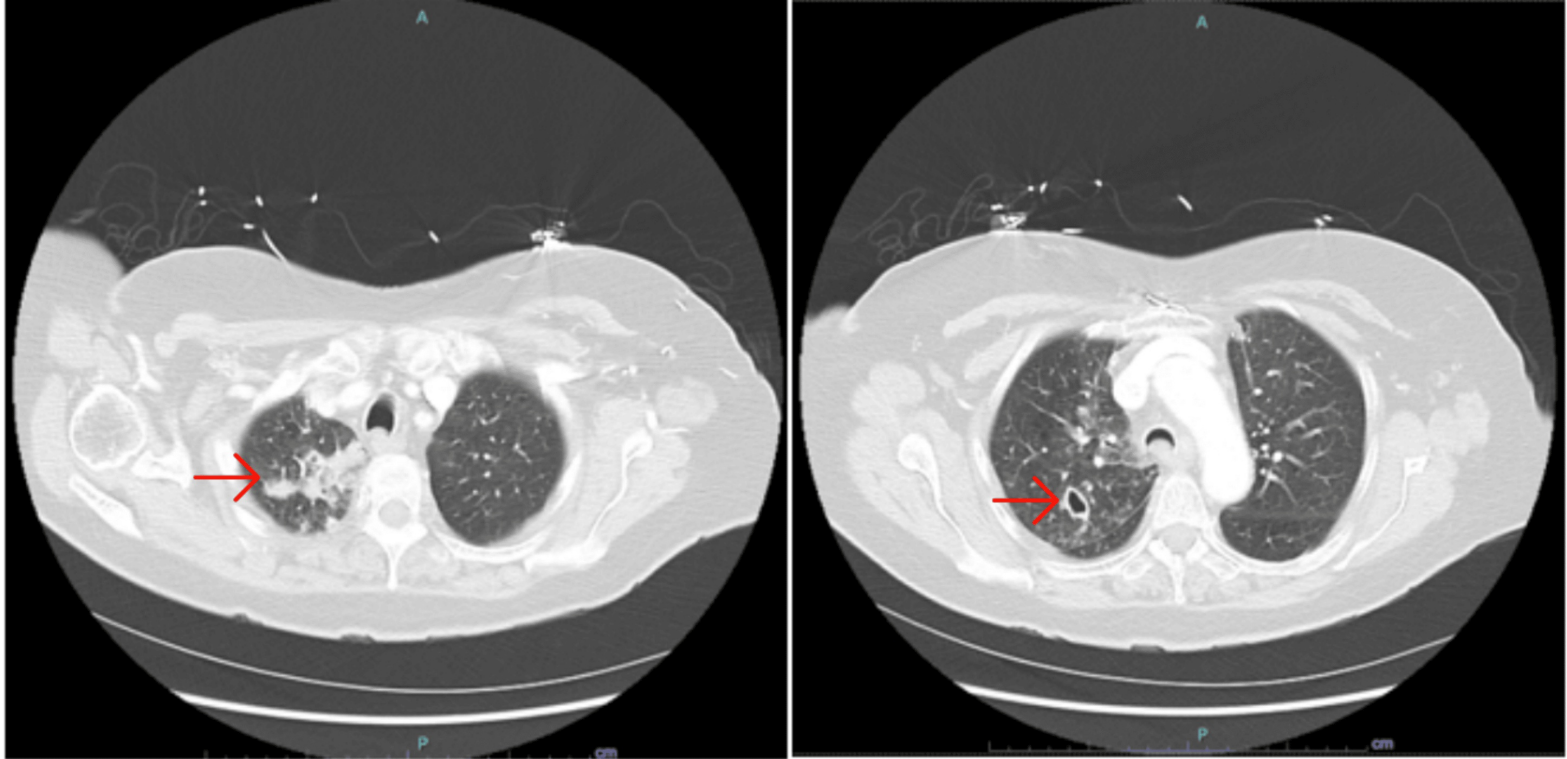
CT chest performed inpatient demonstrated a new thin-walled cavitary lesion in RUL and consolidations in RML and apex. RUL: right upper lobe; RML: right middle lobe

The following day, a CT chest was obtained and demonstrated heterogeneous nodular alveolar and ground glass opacities with a new thin-walled cavity in the right upper lung, and focal areas of airspace consolidation in the right apex and right middle lung suggestive of an atypical infection (Figure 1). Sputum cultures obtained showed 4+ oropharyngeal flora with a moderate Gram-positive cocci burden. Leukocytosis resolved within two days of moxifloxacin treatment, and the patient remained afebrile for the remainder of the hospitalization. Clinically, the patient endorsed improvement in her cough and shortness of breath (SOB) by day three of treatment. Crackles on the initial presentation resolved within the same time frame. Follow-up CT was scheduled four weeks after completion of the antibiotics course for presumed community-acquired pneumonia (CAP) (Figure 2).

On the fifth day of hospitalization, fungal cultures obtained during bronchoscopy prior to hospital admission resulted positive for *Blastomycosis dermatitidis*/*gilchristii*. She was promptly seen by the infectious disease service and started a six to 12 month course of itraconazole with close outpatient follow-up.

## Discussion

Blastomycosis is a fungal infection traditionally seen in the Great Lakes and Ohio River Basin regions. The most common route of infection is inhalation of conidia or fungal fragments from perturbed soil, and most often this manifests with a primary pulmonary infection [2]. Though classically associated with immunocompromised hosts living in endemic regions, blastomycosis is possible in immunocompetent hosts outside this geographical distribution. Symptoms can range greatly from completely asymptomatic infections to fulminant acute respiratory distress syndrome (ARDS) and disseminated disease. Extrapulmonary manifestations of the disease are common and occur in 25-40% of symptomatic infections, and most commonly present as cutaneous, osteoarticular, and CNS blastomycosis [3]. Given the rarity of its occurrence and non-specific initial presentation, delayed diagnosis of blastomycosis (>one month) occurs in more than 40% of cases [3].

This particular case sharply contrasts another case report by Cuddapah et al. In their report, they followed a 36-year-old male patient with a blastomycosis in a non-endemic region. Their patient was able to become asymptomatic for approximately four months using antihistamines, nasal mists, and cough syrups but then eventually had a significant increase in symptoms until a diagnosis was made [4]. Our patient also differs from this case in that their patient showed a fever later [4]. In the study by Alvarez et al., an immunocompetent patient presented with blastomycosis and pleural effusion [5]. This differs from our patient in that our patient did not show pleural effusions. In the study by Bennie et al., the patient also presented with blastomycosis in a non-endemic region. The patient presented genitourinary and osteoarticular involvement. This differs from our patient in that our patient did not show these symptoms [6]. The study by Arnett et al. showed chronic pain and hematuria in two cases of patients with blastomycosis in a non-endemic region [7]. Our patient did not show these symptoms. The study by Randhawa et al. showed cutaneous manifestation in a patient with blastomycosis in a non-endemic region [8]. Our patient did not show these symptoms. The study by Gupta et al. showed periumbilical ulcers in a patient with blastomycosis in a non-endemic region [9]. Our patient did not show these symptoms.

Here, we outlined a case of blastomycosis in a non-endemic region in a patient with no known occupational exposures who did not live or travel to an endemic region. Long-standing COPD likely contributed to her presentation, and she improved clinically with treatment for presumed CAP because azithromycin is anti-inflammatory, but it did not explain why she had this particular pathogen.

## Conclusions

As seen in our patient, a high degree of clinical suspicion is needed to diagnose blastomycosis as it can mimic CAP and other subacute pulmonary infections. It is most often seen in endemic regions, though it should not be excluded for a differential based solely on location. Moreover, additional diagnostic studies should be considered in appropriate clinical contexts. The gold standard for diagnosis remains definitive fungal cultures, but these often take three to four weeks to result. In clinical scenarios where the etiology of the disease is unclear, it is important to supplement diagnostic testing with either blastomycosis urine antigen testing or direct observation of yeast on histopathology studies to ensure timely treatment.
